# Duration from seroconversion to eligibility for antiretroviral therapy and from ART eligibility to death in adult HIV-infected patients from low and middle-income countries: collaborative analysis of prospective studies

**DOI:** 10.1136/sti.2008.029793

**Published:** 2008-07-22

**Authors:** The eligibility for ART in lower income countries (eART-linc) collaboration

## Abstract

**Background:**

Estimation of the number of people in need of antiretroviral therapy (ART) in resource-limited settings requires information on the time from seroconversion to ART eligibility and from ART eligibility to death.

**Objectives:**

To estimate duration from seroconversion to different ART eligibility criteria and from ART eligibility to death in HIV-infected adults in low-income and middle-income countries.

**Methods:**

Participants with documented seroconversion from five cohorts (two cohorts from Uganda, two from Thailand and one from Côte d’Ivoire) were analysed. We used Weibull survival models and Bayesian simulation methods to model true (unobserved) first time of treatment eligibility. We set a consistency constraint so that the mean duration from seroconversion to death was equal to the mean from seroconversion to ART eligibility plus the mean from eligibility to death.

**Results:**

We analysed data from 2072 participants, 16 157 person-years of follow-up and 794 deaths. For the criterion CD4 T-lymphocyte count <200 cells ×10^6^/l, the median duration from seroconversion to ART eligibility was 6.1 years (95% credibility interval 3.3–10.4) for all studies and 7.6 years (95% credibility interval 3.4–15.2) for all but the Thai cohorts. Corresponding estimates for the time from CD4 T-lymphocyte count <200 cells ×10^6^/l to death were 2.1 years (0.7–4.8) and 2.7 years (0.8–8.4). When including all cohorts, the mean time from serconversion to CD4 T-lymphocyte count <200 cells ×10^6^/l and from CD4 T-lymphocyte count <200 cells ×10^6^/l to death represented 66% (38–87%) and 34% (13–62%), respectively of the total survival time.

**Conclusions:**

The duration of different ART eligibility criteria to death was longer than the estimates used in previous calculations of the number of people needing ART. However, uncertainty in estimates was considerable and heterogeneity across cohorts important.

The scale-up of antiretroviral therapy (ART) represents an unprecedented effort to provide access to life-saving drugs to HIV-infected patients in resource-limited settings. The World Health Organization estimates that as a result two million people living with HIV/AIDS in low-income and middle-income countries were receiving treatment at the end of in 2006.^1^ An important question is the coverage of ART in these settings; in other words, what proportion of patients in need of ART is receiving it, and how far away from reaching the goal of universal access are we?

To estimate coverage of ART, information about the untreated natural history of HIV infection in low-income and middle-income countries is needed, especially on the duration from infection (seroconversion) to the point in time when an individual becomes eligible for ART, and from ART eligibility to death.^2^ ^3^ Criteria for treatment eligibility differ between settings and guidelines. For example, the current International AIDS Society-USA guidelines for treatment of HIV infection in adults^4^ recommend that ART should be considered in asymptomatic patients after the CD4 count falls below 350 cells ×10^6^/l and before it declines to 200 cells ×10^6^/l. In contrast, the 2002 WHO guidelines, which are still used in some countries—for example, in South Africa, recommend ART only for patients with WHO stage 4 disease or a CD4 cell count of less than 200 cells ×10^6^/l.^5^ These recommendations were revised in 2003 and now state that, in addition, treatment should also be started in patients with WHO stage 3 and a CD4 count between 200 and 350 cells ×10^6^/l.^6^

Few seroconverter cohorts exist in low-income and middle-income countries, and the number of participants enrolled is relatively small. Collaborative analyses of individual participant data from several studies are useful in this situation.^7^ Recently, Todd and colleagues^8^ estimated time from seroconversion to death in a collaborative analysis of eight seroconverter cohorts from low-income and middle-income countries before ART became available; however, their study did not address time to ART eligibility. We established the eligibility for Antiretroviral Therapy in lower income countries (eART-linc) collaboration with the aim to estimate time from seroconversion to ART eligibility and from ART eligibility to death in HIV-infected adults from lower-income countries.

## METHODS

### Cohort studies

We contacted cohort studies from low-income and middle-income countries of HIV-infected adults with documented last negative and first positive HIV tests and information on the evolution of CD4 counts or clinical stage, and invited them to contribute data to the eART-linc collaboration. Five cohorts, which were established in the 1990s and described in detail elsewhere,^9^^–^13 participated in the current analyses (table 1): the Primo-CI ANRS 1220 cohort^9^ in Abidjan, Côte d’Ivoire; the Rakai District cohort^10^ in rural Uganda; the Masaka District cohort,^11^ also in rural Uganda; the Royal Thai Army conscripts cohort^12^ in Thailand and the blood donors cohort^13^ in Chiang Mai, Thailand. Inspection of the data received showed that the information on CD4 count evolution was limited for the Thai cohorts,^12^ ^13^ and these therefore contributed to estimation of the time from seroconversion to death only. Also, information on clinical stage was available only for the Masaka cohort.^11^

**Table 1:** Characteristics of cohorts and participants included in analyses

		Cohort					Combined
		Côte d’Ivoire (Abidjan)	Uganda (Masaka)	Uganda (Rakai)	Thailand (Royal Thai Army)	Thailand (Chiang Mai)	
Reference		Minga *et al*^9^	Morgan *et al*^32^	Wawer *et al*^33^	Rangsin *et al*^12^	Nagachinta *et al*^13^	–
Study population		Blood donors	Participants in community cohort	Participants in community cohort	Military conscripts	Male blood donors and their female partners	–
Year of start of study		1997	1990	1991	1998	1992	
No of participants included in analyses (%)		253 (100)	338 (100)	411 (100)	233 (100)	837 (100)	2072 (100)
Median (IQR) interval between last negative and first positive HIV-1 test, months		7.5 (3.4–19.1)	12.5 (11.7–24.3)	22.4 (14.6–37.7)	8.5 (5.7–9.5)	50.7 (31.6–69.8)	24.0 (11.8–50.1)
No of women (%)		97 (38.3)	180 (53.2)	256 (62.3)	0 (0)	267 (31.9)	800 (38.6)
No of patients starting ART (%)		79 (31.2)	57 (16.9)	151 (36.7)	0 (0)	0 (0)	287 (13.8)
Mean (SD) age at baseline, years		29.1 (7.1)	32.0 (13.6)	35.8 (2.5)	22.1 (0.9)	28.7 (6.4)	29.9 (8.3)
Median (IQR) baseline CD4 cell count (IQR), cells ×10^6^/l		472 (331–648)	553 (425–743)	409 (235–584)	NA	NA	–
Median (IQR) number of CD4 counts per person		9 (5–14)	5 (2–11)	3 (2–7)	NA	NA	–
Median (IQR) follow up from seroconversion, years		5.4 (5.4–7.6)	4.9 (3.3–7.8)	5.7 (3.8–7.6)	6.0 (5.3–6.7)	6.5 (5.2–9.1)	6.0 (4.4–7.8)

NA, not systematically assessed and therefore not analysed in this study; SD, standard deviation; IQR, interquartile range.

The baseline CD4 count relates to the first measurement after seroconversion.

### Observable and unobservable information

**Figure 1:**
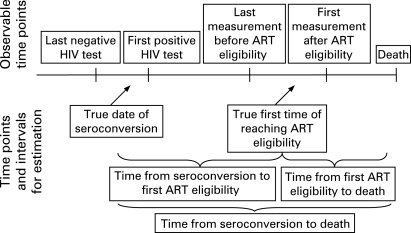
Schematic representation of observed and estimated time points and intervals in the natural history of HIV infection.

Figure 1 shows the observable information and the time points and intervals we set out to estimate. The exact date of reaching treatment eligibility cannot be observed because the relevant criteria, including CD4 counts and clinical stage, are assessed at certain points in time only. Individuals for whom treatment eligibility was observed had two dates: the last date with available information before eligibility was observed and the first date at which available information indicated that ART eligibility had been reached. The true date of treatment eligibility will lie somewhere between these two dates.

### Statistical analyses

We used Weibull survival models^14^ to estimate the duration from seroconversion to various eligibility criteria for ART, from ART eligibility to death or from serconversion to death. The date of seroconversion was calculated as the midpoint between the last documented negative HIV test and the first documented positive HIV test. Follow-up was censored on the date the patient was last known to be alive. In patients who initiated ART, follow-up was censored on the date of starting therapy. We did analyses for various CD4 thresholds (<200 cells ×10^6^/l, <275 cells ×10^6^/l, <350 cells ×10^6^/l) and for the combined criteria WHO clinical stage 3 and CD4 <350 cells ×10^6^/l. In additional multivariable analyses we included gender and age. HIV subtype could not be included because subtype was highly correlated with the country in which the cohort was conducted. For descriptive purposes, we calculated and plotted Kaplan-Meier cumulative incidence estimates for some outcomes for each cohort.

We accounted for differences in survival distributions between cohorts using the hierarchical random effects model assuming proportional hazards. The random effects were assumed to be normally distributed around a common mean. We allowed for two different Weibull distributions, one each for the duration from seroconversion to treatment eligibility and from treatment eligibility to death. For the Thai cohorts, which did not contribute information on treatment eligibility, we modelled the duration from seroconversion to death using a third Weibull distribution. We set a consistency constraint on the mean survival times such that the mean duration from seroconversion to death was equal to the sum of the mean duration from seroconversion to the estimated time of ART eligibility and the mean duration from ART eligibility to death. In an additional analysis we excluded the Thai cohorts.

Estimation of time periods was based on a fully probabilistic approach using simulation methods. Vague prior distributions for parameters of interest were specified by using normal distributions with variances equal to 10^3^. Samples from the posterior distributions for quantities of interest were obtained by Markov Chain Monte Carlo simulations based on 100 000 iterations from which the first 60 000 iterations were discarded. The influence of age at seroconversion and sex on survival was explored in standard proportional hazard Weibull regression models. Age was entered as a continuous variable.

All analyses were performed using Stata (version 9.2, College Station, TX, USA) and WinBUGS^15^ (version 1.4.1, Cambridge, UK). Results are presented as means and medians with 95% credibility intervals (CrI) or as conventional 95% confidence intervals (CI). In Bayesian statistics credibility intervals are commonly used to provide summary information about the probability distribution of a parameter of interest. Similar to 95% confidence intervals, they cover a range in which the true value of the unknown quantity lies with 95% probability.

## RESULTS

**Figure 2:**
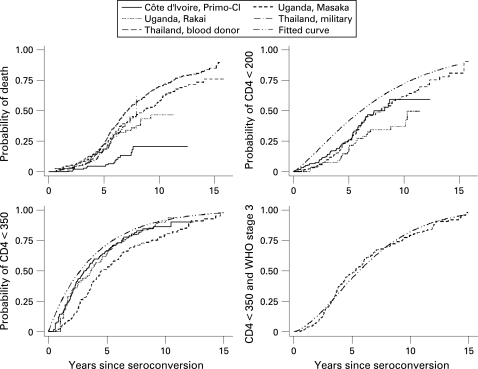
Kaplan-Meier plots of the cumulative probability of death and of reaching different eligibility criteria for antiretroviral therapy.

A total of 2072 adults with documented seroconversion were included in the analyses (table 1). In the African cohorts the follow-up of 294 patients (14.2%) was censored because ART was started. In total, 794 (38.3%) patients died, most of them (762, 96.0%) while not on ART. Figure 2 shows the cumulative incidences from seroconversion to death or to first time ART eligibility was observed for different eligibility criteria. For time to ART eligibility the figure also includes the fitted curves based on the median of the posterior distribution of the common mean of the Weibull parameters. Owing to the difference between the true (but unobservable) time of reaching ART eligibility for the first time, and the first time eligibility was observed, the fitted curves are generally above the observed curves.

Table 2 shows the estimated mean and median years from seroconversion to different ART eligibility criteria and from ART eligibility to death. Means were 1–2 years higher than medians and ranged from 7.6 years from seroconversion to a CD4 count <200 cells ×10^6^/l to 3.9 years for the period from reaching a CD4 count <200 cells ×10^6^/l to death. Corresponding medians were 6.1 years and 2.1 years. For most estimates 95% credibility intervals were fairly wide. The additional analysis excluding the Thai cohorts resulted in intervals that were 0.5 to 1.6 years longer for medians and 0.8 to 2.1 years longer for means, with substantially wider confidence intervals. For the CD4 criteria, the sum of the mean duration from seroconversion to eligibility and the mean duration from eligibility to death gave mean durations from seroconversion to death of 11.3–11.7 years, depending on the eligibility criterion (table 2). Excluding the Thai cohorts resulted in longer periods, with a sum of the means of the two periods ranging from 13.7 years to 14.6 years (table 2). The sum of the means for the combined CD4 and clinical stage criterion, which was estimated using the Masaka cohort data only, was 10.9 years. Note that the medians shown in table 2 cannot simply be added to obtain the median duration from seroconversion to death, only the means add up in this way. To increase applicability to different settings, table 2 also gives time intervals expressed as the percentage of the total time from seroconversion to death.

**Table 2:** Estimated time from seroconversion to different ART eligibility criteria and from ART eligibility to death

Time interval	ART eligibility criteria			
	<200 CD4 cells ×10^6^/l	<275 CD4 cells ×10^6^/l	<350 CD4 cells ×10^6^/l	<350 CD4 cells ×10^6^/l and WHO stage 3
**All cohorts**				
*Seroconversion to eligibility (years)*				
Median (95% CrI)	6.1 (3.3–10.4)	4.2 (1.9–7.9)	2.7 (0.9–6.3)	5.6 (5.0–6.2)
Mean (95% CrI)	7.6 (4.1–12.9)	5.4 (2.5–10.3)	4.0 (1.4–9.2)	6.4 (5.8–7.1)
Percentage*	66 (38–87)	48 (23–75)	34 (12–64)	58 (50–65)
*Eligibility to death (years)*				
Median (95% CrI)	2.1 (0.7–4.8)	4.0 (1.7–8.0)	6.0 (2.9–11.7)	3.0 (2.4–3.8)
Mean (95% CrI)	3.9 (1.4–9.1)	5.9 (2.5–11.7)	7.7 (3.8–15.0)	4.5 (3.5–6.4)
Percentage*	34 (13–62)	52 (25–78)	66 (36–88)	42 (35–50)
**Excluding the two Thai cohorts**				
*Seroconversion to eligibility (years)*				
Median (95% CrI)	7.6 (3.4–15.2)	4.8 (1.9–11.6)	3.2 (1.0–9.7)	5.6 (5.1–6.2)
Mean (95% CrI)	9.4 (4.3–19.2)	6.3 (2.4–15.1)	4.8 (1.5–14.4)	6.4 (5.8–7.1)
*Eligibility to death (years)*				
Median (95% CrI)	2.7 (0.8–8.4)	5.0 (1.6–13.8)	7.6 (3.0–18.3)	3.0 (2.4–3.8)
Mean (95% CrI)	5.2 (1.5–15.9)	7.4 (2.5–20.5)	9.8 (3.8–23.7)	4.5 (3.5–6.4)

*Percentage of duration from seroconversion to death.

CrI, credibility interval.

Heterogeneity of model parameters across cohorts was important. The mean of the random effects standard deviation ranged from 0.8 to 0.92 with lower credibility bounds above 0.4. When including age and sex in the models, estimates of survival were reduced for older people, with hazard ratios ranging from 1.02 to 1.03 per year increase in age with lower bounds of the 95% credibility intervals 1.01 and upper bounds not exceeding 1.05. Analyses comparing women with men gave hazard ratios ranging from 0.91 to 1.09 with wide 95% credibility intervals that always included 1: lower 95% credibility bounds ranged from 0.62 to 0.71, higher bounds from 1.4 to 1.7.

## DISCUSSION

The eART-linc collaboration, which currently includes over 2000 untreated adults from lower-income settings with documented seroconversion, made analyses of individual participant data to estimate the periods from seroconversion to different ART eligibility criteria, and from ART eligibility to death possible.

The collaborative analysis of individual participant data is an important strength of this study: meta-analyses that use individual participant data are considered the gold standard and yardstick for meta-analyses and systematic reviews.^16^ Indeed, in previous reviews of published studies^17^ ^18^ we found that the type of data presented in articles was very heterogeneous, confirming earlier observations on common difficulties with systematic reviews of prognostic^19^ and other observational studies. This approach is also used by the Alpha Network (Analysing Longitudinal Population-based HIV/Aids data on Africa) to analyse patterns of mortality in HIV-infected adults, based on pooled data from several community-based cohort studies.^8^ ^20^ ^21^

The exact date of reaching treatment eligibility can usually not be observed because the relevant criteria, including CD4 counts and clinical stage, are not assessed continuously. One approach to this problem is to take the first date eligibility criteria are met, which may provide a good approximation if the relevant information is collected regularly and frequently. However, this is problematic in the more common situation when intervals between measurements are relatively wide, leading to underestimation of the duration from true ART eligibility to death. In clinical decision-making the date the criteria for ART are documented for the first time is the important date and should lead to the initiation of ART. Of note, many patients in lower-income countries start ART late or very late, with CD4 cell counts well below 200 cells ×10^6^/l.^22^^–^24 However, when estimating the need at the population level, it is the actual date of having reached ART eligibility for the first time that is of interest. In the present analysis we chose an analytical strategy that took into account that only interval information on ART eligibility was available from cohorts. Unsurprisingly, our estimates are higher than published estimates, which took the date when eligibility was documented for the first time as the point of departure. For example, we estimated the median duration from a CD4 count <200 cells ×10^6^/l to death as 2.1 years (2.7 years when excluding the two cohorts from Thailand) and the mean as 3.9 years (5.2 years when excluding the two cohorts from Thailand). Previously published estimates in untreated patients range from 7 months to 22 months (table 3). The distribution of survival times is skewed, which explains that estimates for the mean were higher than those for medians.

**Table 3:** Published estimates of survival from CD lymphocyte counts <200 CD4 cells ×10^6^/l

Study, year of publication	No	Country	Years	Median months of survival (95% CI)
Lawn, 2005^34^	134	South Africa	2002–5	20 (14 to 28)
Costello, 2005^35^	167	Thailand	1993–9	21.6 (19 to 24)*
Badri, 2004^36^	447	South Africa	1992–2001	23.6 (21 to 27)
Schim van der Loeff, 2002^37^	378	The Gambia	1986–97	7 (5 to 9)
Kilmarx, 2000^38^	15	Thailand	1991–8	11 (7 to 15)
French, 1999^39^	78	Uganda	before 1998	9 (7 to 15)
Van der Paal, 2007^28^	147	Uganda	1990–2003	25.7 (21 to 31)

*Men only.

CI, confidence interval.

Mortality patterns after seroconversion have been previously described for several of the seroconverter cohorts included in this analysis.^8^ ^20^ ^25^^–^29 Interestingly, mortality was similar across cohorts, particularly in the first years after seroconversion, despite differences in HIV subtypes. One exception was the cohort from Côte d’Ivoire, which showed lower cumulative mortality than the other four cohorts, despite similar probabilities of observing CD4 criteria for ART eligibility. The lower mortality and morbidity might be explained by under-ascertainment of deaths in this clinic-based cohort or by the early introduction of cotrimoxazole prophylaxis, in 1994, by the Centre National de Transfusion Sanguine in Abidjan.^9^ Indeed, two clinical trials from Côte d’Ivoire and one from Senegal showed a substantial reduction of mortality: the combined relative risk was 0.69 (95% confidence interval 0.55 to 0.87).^30^

About five years after seroconversion mortality was higher in the Thai studies and, particularly, in the Chiang Mai blood donor cohort.^13^ The inclusion of paid blood donors of lower socioeconomic strata might explain the higher mortality in this cohort. There is debate on whether HIV subtype (predominantly subtype E in Thailand) influences survival.^8^ The additional analysis excluding the Thai studies therefore resulted in longer duration estimates. In addition to reporting time intervals in years, we therefore also expressed intervals as the percentage of the total time from seroconversion to death. This should facilitate the application of results to populations with different background and HIV-related mortality. We found that depending on the ART eligibility criteria considered, 34% to 66% of the mean duration from seroconversion to death occurs after having become eligible for ART.

Our analysis has several limitations. The number of included cohorts and the number of seroconverters was small and information on treatment eligibility was available for only three of the five cohorts. We used a simple midpoint approximation to estimate the date of seroconversion, despite the fact that in some cohorts intervals between tests were long. Estimation of model parameters became difficult when allowing uncertainty about the date of seroconversion as well as the time of reaching ART eligibility. Also, midpoint approximation is common practice in analyses of survival time from seroconversion to death. We analysed all-cause mortality and could not distinguish between HIV-associated mortality and other causes of death. We used parametric survival models and thus made assumptions about the shape of the survival distributions that may not always be appropriate.

In conclusion, estimation of the number of people in need of ART in resource-limited settings should be based on figures for survival from ART eligibility to death that are somewhat higher than those used previously,^2^ and support the recent updates to the Joint United Nations Programme on HIV/AIDS (UNAIDS) Spectrum projection software.^31^ Finally, we would welcome additional studies in the eART-linc collaboration in order to obtain more precise and more widely applicable estimates in future analyses. Interested investigators are invited to contact us at eART-linc@ispm.unibe.ch.
